# Eye Movement Patterns in Russian-Speaking Adolescents with Differing Reading Comprehension Proficiency: Exploratory Scanpath Analysis

**DOI:** 10.3390/jintelligence12110112

**Published:** 2024-11-05

**Authors:** Alexandra Berlin Khenis, Maksim Markevich, Anastasiia Streltsova, Elena L. Grigorenko

**Affiliations:** 1 Center for Cognitive Sciences, Sirius University of Science and Technology, Sirius 354340, Russia; berlin-henis.aa@talantiuspeh.ru (A.B.K.); markevich.mo@talantiuspeh.ru (M.M.); streltsova.av@talantiuspeh.ru (A.S.); 2 Department of Psychology, 126 Heyne Bldg, University of Houston, Houston, TX 77204, USA; 3 Yale Child Study Center, 230 South Frontage Road, New Haven, CT 06520, USA

**Keywords:** eye tracker, scanpath analysis, reading comprehension, text reading, eye movement patterns

## Abstract

Previous research has indicated that individuals with varying levels of reading comprehension (often used as a proxy for general cognitive ability) employ distinct reading eye movement patterns. This exploratory eye-tracking study aimed to investigate the text-reading process in adolescents with differing reading comprehension, specifically examining how these differences manifest at the global eye movement level through scanpath analysis. Our findings revealed two distinct groups of scanpaths characterized by statistically significant differences in eye movement parameters. These groups were identified as “fast readers” and “slow readers”. Both groups exhibited similar oculomotor performance during the initial reading. However, significant differences emerged when they reread and revisited the text. Notably, these findings align with prior research conducted with different samples and languages, although discrepancies emerged in saccade amplitude and first-pass reading behavior. This study contributes to the understanding of how reading comprehension levels are reflected in global eye movement strategies among adolescents. However, limitations inherent in the experimental design, particularly the potential influence of the task on reading patterns, warrant further investigation. Future research should aim to explore these phenomena in more naturalistic reading settings, employing a design specifically tailored to capture the nuances of spontaneous reading behavior.

## 1. Introduction

Reading comprehension plays a crucial role in academic achievement in general and in adolescence in particular, when reading comprehension substantiates reading for meaning, serves as a proxy for general cognitive ability, and becomes a significant factor that contributes to overall success in work and life. Schooling presumes not only the mastery of basic language skills, including word decoding, vocabulary, morphological and syntactic awareness, but also of higher-level cognitive skills, i.e., making inferences and drawing conclusions from the text, analyzing text structures, substantiating judgments about the text content (Oakhill et al. 2003; Perfetti et al. 2005; Rapp et al. 2007; Kendeou et al. 2016). Proficient reading skills allow for the usage of written materials to progress in various academic fields. Moreover, adept reading skills are highly important for learning in digital settings, and they have become both hugely popular and essential for knowledge acquisition (OECD 2023: Insights and Interpretations). Since the reading proficiency expectations in adolescence exceed basic reading mastery, those who experience reading comprehension difficulties inevitably suffer the consequences in multiple academic domains (Kraal et al. 2018).

Simple View of Reading (SVR; Gough and Tunmer 1986; Hoover and Gough 1990) considers reading efficiency a product of decoding and linguistic comprehension. Decoding is the ability to recognize individual written words and translate graphic symbols into sounds using the alphabetic principle (Perfetti 1985; Adams 1990; Lee et al. 2022). Linguistic comprehension refers to the set of skills needed to interpret connected discourse. This set includes listening comprehension, or the ability to interpret spoken language (Talwar et al. 2021), and vocabulary knowledge (Braze et al. 2016), which consists of vocabulary breadth and depth (Protopapas et al. 2013). Although failures in decoding or linguistic comprehension can cause reading impairment or language impairment, respectively (Gough and Tunmer 1986), these components are highly interrelated, and each component alone is not sufficient for the deficit onset (Quinn and Wagner 2018). Other predictors of reading efficiency that were not part of the initial SVR model include working memory (Cain et al. 2004; Baddeley et al. 2009), background knowledge (Spires and Donley 1998; Evans et al. 2002), and inference (Kendeou et al. 2008; Oakhill and Cain 2012) and reasoning (Pammer and Kevan 2007) skills.

Reading comprehension at a psychophysiological level can be explored through the analysis of eye movements (e.g., Mézière et al. 2023, 2024; Gattei et al. 2018; Jian 2022). The analysis of eye movement patterns provides an in-depth understanding of the complex process of text comprehension. Specifically, eye movement characteristics have been demonstrated to vary across differences in readers’ linguistic abilities, componential reading skills, and reading comprehension proficiency (Rayner 1998; Berzak and Levy 2023; Southwell et al. 2020; Wilkinson and Payne 2006; Duggan and Payne 2009; Strukelj and Niehorster 2018). The analysis of the dynamics of oculomotor characteristics, such as fixations and saccades, while focusing on specific elements of the text (words or sentences) allows us to study the specifics of these text elements’ processing (Magyari et al. 2020; Bråten et al. 2015; Chen et al. 2023) at different levels of language and reading skills (Kuperman et al. 2018; Korneev et al. 2020; Parshina et al. 2021).

In the literature, there are strong indicators that eye movements are sensitive to high-level processes occurring during text reading (Rayner et al. 2012; Schotter et al. 2014; Faber et al. 2018). This observation is primarily supported by studies examining the relationship between specific patterns of reading and reading comprehension (Duggan and Payne 2009; Strukelj and Niehorster 2018; Randall et al. 2014; Hyönä et al. 2002; Inhoff et al. 2016; Metzner et al. 2017). For example, skim reading expresses a smaller number of fixations; moreover, these fixations are shorter than in typical reading. Most often, the use of skim reading is associated with the task of extracting specific information from the text in a shorter time compared to typical reading and is indicative of superficial reading comprehension (Duggan and Payne 2009; Rayner et al. 2012; Schotter et al. 2014; Strukelj and Niehorster 2018). Similar to skim reading, mind-wandering reading is also associated with fewer fixations, but the duration of these fixations, contrary to skim reading, is longer compared to typical reading. Mind wandering is also associated with more shallow reading comprehension (Faber et al. 2018; Randall et al. 2014). However, with inattentive reading, readers can correct comprehension deficits during reading by changing strategies: attention is specifically directed to the parts of the text where comprehension loss occurred (Hyönä et al. 2002; Inhoff et al. 2016; Metzner et al. 2017).

Meanwhile, unambiguous, consistent links between reading comprehension strategies and specific eye movements still must be established. For example, some studies indicate a correlation between the number of regressions (e.g., returns to specific text elements) and improving reading comprehension when reading text (Hyönä et al. 2002; Inhoff et al. 2016; Schotter et al. 2014), while others show no correlation (Christianson et al. 2017). Moreover, the same oculomotor characteristics may be indicative of different reading-related processes. For example, longer fixations have been reported to be characteristic of distracted reading (mind-wandering), which, in turn, is a predictor of poorer comprehension (Faber et al. 2018). However, corrective action, ultimately leading to improved comprehension, is also associated with longer fixations (Frazier and Rayner 1982). Longer fixations are observed when texts appear difficult for the reader (Rayner et al. 2006). Therefore, the existing ambiguity in interpreting eye movement characteristics while reading does not allow us to clearly identify which specific eye movement parameters reflect better comprehension. The discrepancies presented above relate to the type of analysis at the level of local measurements, where each parameter is presented for analysis in an averaged form at the level of the text area or element of interest, which often includes a letter, a single word, or part of a sentence. However, extracting meaning from text by a reader is a continuous process of perception without highlighting or focusing on individual words. Therefore, one possible way to study the process of reading comprehension could be to turn to a global analysis of the entire pattern of reading text.

Yet, the analysis of eye movements while reading whole texts requires specific methods of analysis. Currently, there are several alternative ways of analyzing oculomotor characteristics: path analysis (Inhoff et al. 2016), random forest analysis (Matsuki et al. 2016), and scanpath analysis (Von Der Malsburg et al. 2015). Scanpath analysis has several advantages that allow it to be considered promising in research into whole-text reading (Von Der Malsburg and Vasishth 2011, 2013; Von Der Malsburg et al. 2015). The main aim of this method is to capture eye gaze behavior at the sentence or discourse level, which provides a more holistic view of the overall picture of gaze behavior during reading. For example, when regressive eye movements are of interest (i.e., when processing ambiguous sentences), scanpaths capture not only the presence of regression but also its target (e.g., the beginning vs. some other part of a sentence) as well as its type (e.g., singular short regressions vs. multiple longer rereadings).

The method of scanpath analysis has been shown to be effective in studies searching for predictors of proficient reading comprehension. For example, recent work by Mézière et al. (2024) has shown that differences in eye movement strategies provide information about the level and quality of reading comprehension. There are also examples of applying this type of analysis to the identification of differences in gaze trajectory between readers with different language abilities and different language experiences. Parshina et al. (2022a) used scanpath analysis to investigate reading in monolingual Russian adults, children, and bilingual speakers (native speakers of one of the minority languages of the Russian Federation who also spoke Russian as their second language), and adult learners of Russian. The four-group analyses identified three most typical reading patterns: (1) “fluent” reading, characterized by regular left-to-right eye movements, short fixations, few regressions, and a high proportion of skips; (2) “intermediate” reading, characterized by longer fixations, short regressions to individual words, and fewer skips; and (3) “beginner” reading, characterized by the longest fixations, frequent rereading of the whole sentence, and almost no skips at all. As expected, the four groups of readers differed in their use of these three strategies. While adult monolinguals tended to use a “fluent” scanning strategy, children, native speakers of a minority language, and Russian language learners were more likely to use “intermediate” and “beginner” strategies. The findings suggest that the use of different reading strategies may reflect the level of reading comprehension and, therefore, may be a useful predictor of reading comprehension proficiency.

The vast majority of eye-tracking studies examining eye-movement patterns during reading have been conducted using samples of native speakers of English (Wilkinson and Payne 2006; Duggan and Payne 2009; Mézière et al. 2023). Interestingly, although reading strategies seem to be relatively universal (e.g., skimming and scanning), it appears that language characteristics can influence the type of eye movement patterns typical of a given reading strategy. Thus, studies focused on other languages, for instance, German (Von Der Malsburg et al. 2015), Swedish (Strukelj and Niehorster 2018), Chinese (Meng-Jung Tsai et al. 2022), and Russian (Parshina et al. 2022a, 2022b), are instrumental for determining the impact of linguistic characteristics on reading in general and reading strategies in particular.

The aim of this exploratory study was to examine characteristic features of scanpath profiles during reading among Russian adolescents, with a focus on how specific eye movement patterns are related to reading comprehension. To achieve this aim, we (1) identified groups of adolescents with different reading comprehension levels based on their eye movement patterns (scanpaths) while reading and (2) investigated whether the reading comprehension level influenced the probability of usage of specific eye movement patterns (one of the possible scanpaths).

This study adopts an exploratory approach; however, based on previous research (Hyönä et al. 2002; Vorstius et al. 2014; Inhoff et al. 2016), we hypothesized that adolescents with varying reading comprehension proficiency would exhibit different scanpaths during reading. Specifically, we anticipated a higher reading time per paragraph (the text was divided into 12 paragraphs), a higher number and longer duration of fixations per word, and a shorter amplitude of saccades for readers with poorer reading comprehension skills. Additionally, consistent with findings from previous studies (Hyönä et al. 2002; Parshina et al. 2022b), we expected to observe a higher frequency of regressions and longer rereading time in adolescents with lower reading comprehension proficiency.

## 2. Materials and Methods

The present research was granted ethical clearance by the Sirius University of Science and Technology’s Ethics Committee (Protocol from 31 August 2021). Written informed assent and consent were procured from the participating children and their legal representatives, respectively. This research was conducted in strict compliance with the tenets enshrined in the Declaration of Helsinki. Participant data were collected as part of the pilot phase of a research project titled “Literacy as a Foundation of Knowledge Economy: A Neuroscientific Approach.” This project encompasses a total of five psychophysiological studies, during which both EEG and eye-tracking data were recorded. The findings from a pilot experiment from one of these studies have been reported (Markevich et al. 2024).

Participants completed both behavioral and psychophysiological blocks. The behavioral block assessed reading skills, evaluating reading comprehension, memory, decoding, and vocabulary (see Section 2.2 for a detailed description and Figure S1 for task sequence). The psychophysiological block involved participants completing recall and true–false tasks while using the eye-tracker. A break of approximately one hour separated the two blocks.

### 2.1. Participants

We recruited 34 participants. Due to technical problems, five participants were unable to complete the eye-tracking protocol, and one participant was excluded because of poor eye-tracking data quality. The ultimate sample consisted of 28 adolescents (22 females) with an age range spanning from 13 to 17 years (mean age = 15.17, SD = 1.56). All participants possessed normal or corrected-to-normal vision and had no prior history of psychological disorders, language deficits, substance abuse, or neurological injury. Descriptive participant data, including their demographic characteristics, reading habits, and language environment, are provided in Supplementary Tables S1 and S2.

Employing G*Power 3.1 (Faul et al. 2009), a sensitivity analysis demonstrated that, given our sample size (n = 28) and assuming a power of 0.80, the conducted analysis exhibited adequate sensitivity to register a medium effect size of 0.39 using linear multiple regression with two predictors.

### 2.2. Assessment of Reading Skills

According to the SVR model, reading comprehension is composed of several components. Several techniques were used to assess these components. All assessments were developed for the research project “Literacy as a Foundation of Knowledge Economy: A Neuroscientific Approach” mentioned above. The assessments are listed below.

#### 2.2.1. Reading Comprehension Task

Stimuli. The behavioral assessment of reading comprehension consists of three texts (~650–750 words per text) with 10 questions/assignments for each (i.e., 30 questions overall). The three texts are mixed-type texts and consist of a set of elements in both continuous (e.g., newspaper reports, essays, novels, short stories, reviews, letters) and non-continuous (e.g., lists, tables, graphs, diagrams, advertisements, schedules, catalogs, indices) formats. The reading comprehension task matches the PISA 2018 framework (OECD 2019). The distribution of item type is the same for the three texts: (1) two items assess the skill of locating the relevant information; (2) five items assess the skill of understanding the content provided; and (3) three items assess the skill of evaluating the content.

Procedure. Participants silently read three non-fiction texts and completed the corresponding assessment items using a web platform installed on laptops or touchscreen tablets.

#### 2.2.2. Wordspan and Pseudowordspan Task

Stimuli. In the Wordspan and Pseudowordspan tasks, nouns meeting the following criteria were utilized: five to seven letters and two syllables in length and with a frequency from 20 to 100 ipm (median 40.2) according to the dictionary of Lyashevskaya and Sharov (Lyashevskaya and Sharov 2009). The list of stimuli did not include emotionally colored words (e.g., war, death), animal or bird nouns (e.g., cat, woodpecker), abstract nouns (e.g., fate, business), or nouns associated with gender (e.g., lady, man).

Procedure. The participants were presented with these tasks while sitting at a computer. The stimuli were presented auditorily; the participant was required to repeat all words with the condition that the last word presented should not be repeated first. The presentation of the stimuli was as follows: a list of two words was played, and then the participants repeated what they remembered. To follow, another word pair was presented, and the participant was asked to repeat these two words. After the first two pairs, the number of words increased by one in every other combination; the final two combinations consisted of eight words. Overall, there were 14-word combinations (i.e., 2, 2; 3, 3; 4, 4; 5, 5; 6, 6; 7, 7; 8, 8 words). Real words were presented first, and pseudowords second. The procedure for word and pseudoword presentation was identical, yet there were fewer (10) pseudoword combinations, and the last combination included only six pseudowords (2, 2; 3, 3; 4, 4; 5, 5; 6, 6 pseudowords). Prior to the data collection initiation, all participants were presented with practice combinations (one two-item and one three-item combination) both for words and pseudowords parts to introduce the procedure, the volume, and the stimuli presentation interval.

#### 2.2.3. Word and Pseudoword Decoding Task

Stimuli. Word and pseudoword decoding skills were assessed using previously developed tasks modeled after the WRAT-3 word reading subtest (Wilkinson 1993). The utilized word list includes 42 words of increasing complexity controlled by syllabic difficulty, word length (one–six syllables), and word frequency. The pseudoword decoding task includes a list of 20 pseudowords of increasing complexity, also controlled by syllable structure corresponding to phonotactic and orthographic rules of the Russian language and pseudoword length (two to five syllables). For detailed information, see Rakhlin et al. (2023).

Procedure. The assessor presented participants with a sheet of paper with real words upside down. After the command “Start!”, the participant was instructed to turn the sheet over while the assessor pressed “Start” on the stopwatch. The participants read the words out loud one by one at their own pace. The assessor marked the words read with mistakes. After the participant reads the last word, the assessor pressed “Stop” on the stopwatch and marked the time in seconds on the evaluation form. The number of words and pseudowords read correctly and the time it took were used in the subsequent analyses.

#### 2.2.4. Synonym Task

Stimuli. The synonym task is part of a newly developed literacy assessment for the Russian language (Logvinenko et al. 2021). The stimuli consist of 50 words denoting an object, phenomenon, action, or feature of the subject and contain nouns, adjectives, and verbs of one of the three vocabulary tiers. Tier One represents basic vocabulary; Tier Two includes high-frequency words that might have multiple meanings; and Tier Three includes field-specific vocabulary. The words are arranged in ascending order of difficulty. Less difficult stimuli are represented by more frequently used and concrete words, while more difficult stimuli are represented by more rarely used and abstract words. Answers were selected using the *Dictionary of Russian* synonyms and expressions similar in meaning (Abramov 2008) and the Web Dictionary of Russian synonyms (https://classes.ru/ (accessed on 1 March 2019)). In addition, synonyms were collected from a focus group of 11 neurotypical adults. Synonyms from the dictionaries and focus group responses were evaluated for synonymy by four experts with backgrounds in philology/linguistics.

Procedure. This assessment was presented orally. The assessor read the word aloud, and the participant was instructed to identify its proper synonym. A maximum of 10 s and two attempts were allowed per word. All the correct answers were recorded and summed up.

### 2.3. Experimental Procedure

The experimental session began with a briefing during which the participants were informed about the tasks and procedures. Before recording, an individual calibration and validation process of equipment for each participant was performed to ensure eye-tracking registration accuracy. During this procedure, participants were instructed to track a moving point on the computer screen. The point had nine designated stops strategically placed to cover the entire screen. Additionally, during the experiment, participants encountered drift correction points positioned between stimuli. They were instructed to fixate their gaze on these central points. This procedure served as a valuable method for correcting and ensuring the accuracy of the calibration. If poor detection of the drift correction point was noticed, the calibration was repeated. The total time for the experiment was approximately 45 min.

**Figure 1:**
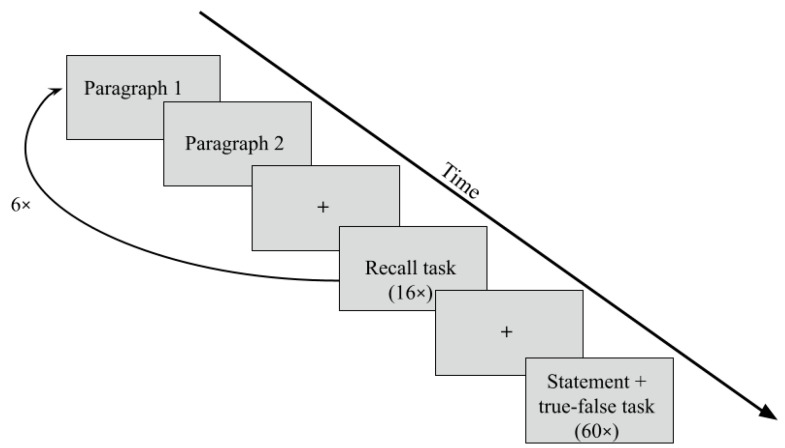
A scheme of the experimental design. The experiment included 12 expository paragraphs. The recall task included 16 words after each of the two paragraphs (96 words in all). The recall task was repeated 6 times. The true–false task included 30 true and 30 false statements (60 statements in all). The total time for the experiment was approximately 45 min.

Recall and True–False tasks. The experiment consisted of several steps (see Figure 1). The participants were presented with 12 paragraphs in between the readings in which they completed the recall task (the participants were required to remember whether a presented word had been mentioned in the two previous paragraphs). The participants had to press one of the two buttons to indicate whether the item was contained in the paragraph they read or not. This process was repeated 6 times, with each block containing a different paragraph. After reading all the paragraphs and completing the recall tasks, the participants moved on to the second block of the study, which involved the true–false task. In this task, they were presented with 60 statements and asked to determine whether each statement was consistent with the information presented in the text previously. The same buttons were used for the true–false task. The time to read the texts and complete both tasks was not limited. Before the start of the main task, participants were asked to attend a training session, where they could work through all the stages and mechanics of the experiment in a shortened version.

Stimuli. The participants were presented with an expository text divided into 12 paragraphs containing up to 7–10 single-spaced lines each (75–111 words). Apart from reading the paragraphs, this subtest contained two tasks: the recall task and the true–false task. The recall task was presented once after every two consecutive paragraphs, for a total of six times (see Figure 1). The participants were presented with a noun that was either used in the two previous paragraphs (eight items = targets) or was unrelated (eight items = distractors) to them (sixteen items overall). Recall targets were used once in the text. The unrelated items were matched for frequency (*Mipm* = 5.2) and syllable length (the number of syllables for the recall items was three). They were also from the same semantic class but not synonymous with the target. The true–false task was presented after the participants had read all the paragraphs and completed the recall task. The participants were presented with 30 true and 30 false statements based on the paragraphs presented earlier. All statements were plausible and parallel in structure.

### 2.4. Apparatus and Recordings

The experiment took place in a soundproof room, and participants were seated approximately 75 cm away from the display screen. A headrest was provided to minimize head movements. All stimuli were presented in the black, 22-point Arial font on a light gray background programmed in Experiment Builder (SR Research Ltd., Ottawa, ON, Canada). We used a Lenovo Legion 144 Hz monitor (resolution: 1920 × 1080 pix) controlled by a computer. Eye movements were recorded with an EyeLink 1000+ eye tracker (SR Research, Toronto, ON, Canada). The right eye was tracked at a 1000 Hz rate.

### 2.5. Data Pre-Processing

The eye-movement data were pre-processed in Data Viewer (SR Research, Toronto, ON, Canada). Based on visual inspection of the data, we excluded participants and trials with poor calibration from this analysis. As a result, data from 9.52% of trials were excluded. Each word was marked as a separate area of interest, with punctuation-related areas excluded from the analysis. For each participant, fixations less than 60 ms and longer than 800 ms were excluded. The analysis of eye movements included only data related to paragraph reading.

### 2.6. Data Analysis

The statistical analyses were performed in R software version 2023.03.01 (R Core Team 2023) using the lme4 (Bates et al. 2015), tidyverse (Wickham et al. 2019), mclust (Scrucca et al. 2023), MASS (Venables and Ripley 2002), *patchwork* (Pedersen 2024), sjPlot (Lüdecke et al. 2024), and scanpath routines (Von Der Malsburg 2018). The data and analysis code for this study can be obtained by contacting the first author.

#### 2.6.1. Behavioral Data

Subtest scores. As we expected the reading comprehension scores, the age of the participants, and other subtest scores to be associated, Pearson’s correlations were calculated. All behavioral variables scores were centered and scaled. Centering refers to the process of subtracting the mean from each score, which adjusts the data to have a mean of zero (Johnson and Wichern 2007). Scaling involves dividing each centered score by its standard deviation, resulting in a standard deviation of one (Johnson and Wichern 2007). This standardization facilitates comparisons across variables by ensuring they are on a similar scale, thus minimizing the influence of differing units or variances.

Accuracy. A logistic mixed-effects model was utilized with the glmer command in R. The centered and scaled scores of the Reading Comprehension Assessment (max score of 60) were included in the model as the fixed factor. Age was also included as a fixed factor to control for potential age-related differences in performance. Random intercepts for participants and trials were included as random factors to account for individual differences among participants and potential variability across paragraphs. The intercept of the model included the mean-centered values of reading comprehension score and age. The structure of the models was the same for both the recall task and the true–false task.

Response time. The reaction times (RTs) per response were assessed. The logarithmic transformation was used to standardize the right-skew distribution of the RTs. All observations that fell outside of 3.0 SD from the mean were considered outliers (2.34% for the recall task and 1.85% for the true–false task).

To investigate the connection between reading comprehension and RTs, linear mixed-effects models were employed with the same predictors as in the accuracy models. The centered and scaled score of the Reading Comprehension Assessment was included in the model as the fixed factor. Age was also included as a fixed factor to control for potential age-related differences in RTs. Random intercepts for participants and trials were included as random factors to account for individual differences among participants and potential variability across paragraphs. The intercept of the model included the mean-centered values of reading comprehension score and age.

#### 2.6.2. Eye-Tracking Data

**Figure 2:**
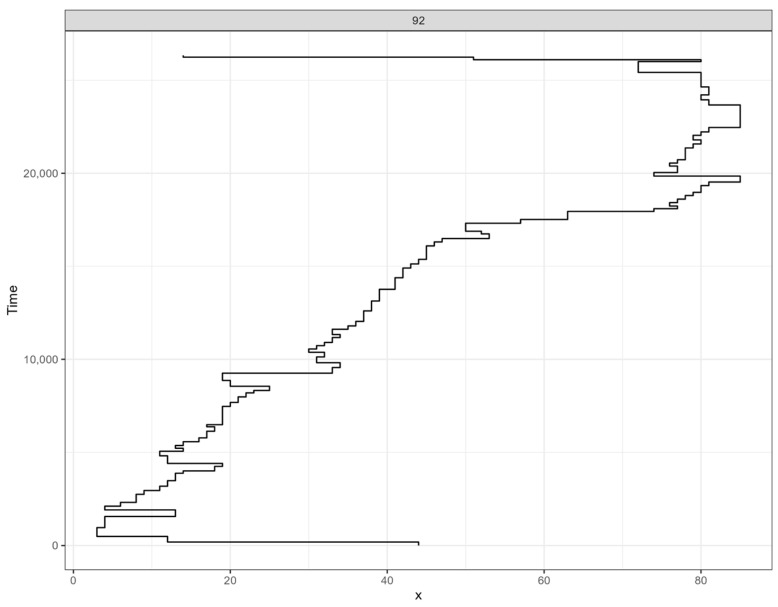
An example of scanpath visualization for one participant while reading a paragraph. The X-axis represents the word number, and the Y-axis represents the reading timeline. The scanpath data represents the pattern of eye movements while reading the paragraph. Horizontal lines in the graphs represented saccadic movements, while vertical lines represented fixation duration.

To answer our research aim, we conducted several stages of oculomotor activity analysis. First, we identified specific groups of scanpaths similar to the analysis described in the articles by Parshina et al. (2022b) and Mézière et al. (2023). First, the scanpath (eye movement patterns) for each participant was calculated for each paragraph for visual evaluation (see Figure 2, for example). Second, we calculated the differences between scanpaths for each paragraph, considering both spatial distance and fixation duration. Then, we used the method of multidimensional scaling to create maps representing the similarities and differences in scanpaths. Each point on the map corresponds to a participant’s eye movement pattern for a specific sentence. Scanpaths located closer together on the map indicate similar reading patterns, while those farther apart reflect more divergent reading patterns. We followed the approach of Ziubanova et al. (2023) for cluster division. We consistently observed two clusters in the data, with a range of two to nine. To minimize random variation, we analyzed the data using two clusters for all trials. While individual reading fluency can vary, leading to different clusters in scanpaths, we expect each participant to exhibit a dominant eye movement strategy, reflected in the prevalence of one cluster in their data.

After the identification of two groups of scanpaths across all trials and participants, we compared oculomotor characteristics between these clusters. For this, we used a linear mixed-effects model. The following oculomotor parameters were treated as dependent variables: the average duration of fixation in a word, average number of fixations in a word, amplitude of saccades, total number of regressions, total time spent at a word, total reading time for the paragraph, skipping rate, rereading time after first-pass reading, number of regressions after first-pass reading, and gaze duration per word during first-pass reading. For each eye-movement parameter, we built a separate model. For each model, the number of clusters was included as a fixed effect. The random intercept of participants and trials were used as random factors. Each model included dummy-coded categorical fixed effects (cluster 1 coded as 0, cluster 2 coded as 1), so the intercept of each model included values for cluster 1.

To investigate whether reading comprehension proficiency predicts the choice of a specific reading strategy, we used a logistic mixed-effects model. In this model, the dependent variable was the cluster and the scaled score of the reading comprehension task, and age were used as fixed factors. The model also included a random intercept for participants and texts as random effects. The intercept for the model included the mean-centered values of reading comprehension and age.

## 3. Results

### 3.1. Behavioral Results

#### 3.1.1. Correlations

Reading comprehension scores positively correlated with all assessment scores and did not correlate with the participants’ age (see Table 1).

#### 3.1.2. Accuracy

**Figure 3:**
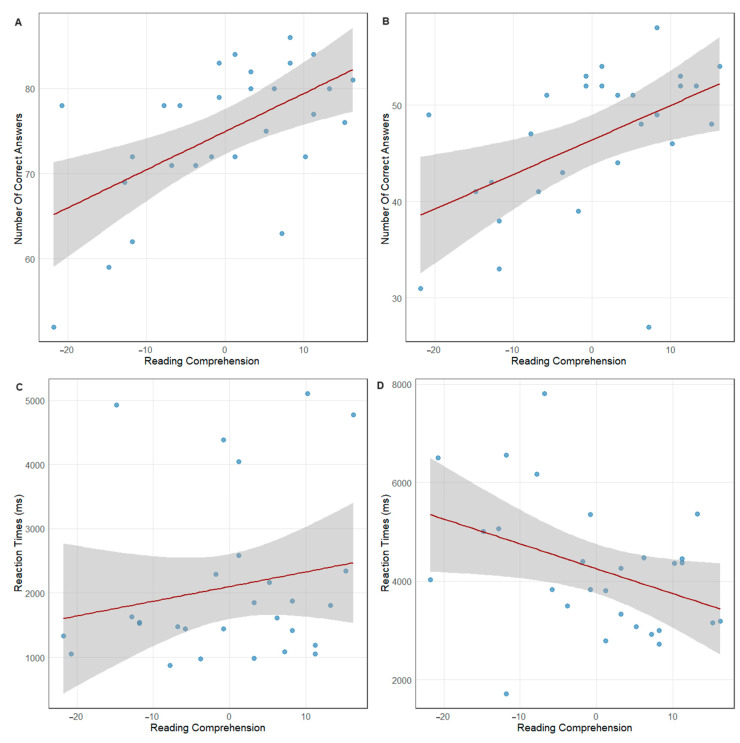
Correlation plots for the reading comprehension scores and (**A**) number of correct answers in the recall task; (**B**) number of correct answers in the true–false task; (**C**) reaction times in the recall task; (**D**) reaction times in the true–false task. The shaded band represents one standard error of the line of the best fit (trendline). Each dot corresponds to an individual participant.

Recall task: The fixed effect of the reading comprehension was statistically significant, where b = 0.260, SE = 0.081, z = 3.195, *p* = .001, indicating that the higher the RC, the higher the number of correct answers (see Figure 3A). The fixed effect of the participant’s age was not statistically significant (see Supplementary Table S3).

True–false task: The fixed effect of the reading comprehension was statistically significant, where b = 0.409, SE = 0.156, z = 2.633, *p* = .008, indicating that the higher the RC, the higher the number of correct answers (see Figure 3B). The fixed effect of the participant’s age was not statistically significant (see Supplementary Table S3).

#### 3.1.3. Response Time

Recall task: The fixed effect of the reading comprehension and participant’s age was not statistically significant (see Supplementary Table S4).

True–false task: The fixed effect of the reading comprehension was statistically significant, where b = −587.22, SE = 250.73, t(27.79) = −2.342, *p* = .027 (see Figure 3D). The fixed effect of the participant’s age was not statistically significant (see Supplementary Table S4).

### 3.2. Eye-Tracking Results

**Figure 4:**
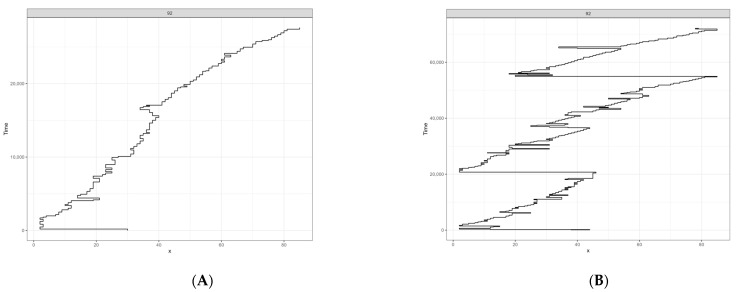
Examples of typical eye movement patterns during paragraph reading from two clusters we identified based on eye movement parameters during reading. On the left, a scanpath from cluster 1 is presented (**A**), while on the right, there is a scanpath from cluster 2 (**B**). The X-axis represents the word number information, and the Y-axis represents the reading timeline. The scanpath data represent the pattern of eye movements while reading the paragraph. Horizontal lines in the graphs represented saccadic movements, while vertical lines represented fixation duration.

The descriptive statistics (mean and standard deviation) for eye movement of the clusters are presented in Table 2. Linear mixed-effects models were used to assess the difference between clusters of scanpaths in eye movement parameters. The results of the analysis for each model are presented in Table 3, including the b coefficient, confidence intervals, and *p*-value. Statistically significant differences between scanpath clusters were detected. Reading patterns from cluster 2 can be described by a longer average duration of fixation (Group 2: t = 4.17, *p* < .001), longer total reading time per word (Group 2: t = 5.54, *p* < .001), higher average number of fixation per word (Group 2: t = 4.79, *p* < .001), higher total number of regression (Group 2: t = 2.74, *p* < .006) and longer total reading time per paragraph (Group 2: t = 6.68, *p* < .001) in comparison with reading patterns from cluster 1. Figure 4 presented examples of typical eye movement patterns during paragraph reading from two clusters we identified based on our data.

After identifying two clusters of scanpaths during reading, we used a logistic mixed-effects model to investigate whether the level of reading comprehension predicts the usage of a specific type of scanpath from one of the two clusters. Table 4 presents the results of the analysis, including data on b coefficients, standard errors, and *p*-values. The results indicate that the higher the reading comprehension level, the more likely an individual is to use scanpaths from cluster 1 (*p* < .0001) and the less likely they are to read using scanpaths from cluster 2 (*p* < .0001). At the same time, age did not appear to be a significant predictor in any of the models.

## 4. Discussion

### 4.1. Behavioral Data Results

To recap, we observed positive correlations between reading comprehension and several indicators of reading performance, such as decoding, vocabulary, and verbal working memory. We also discovered that response accuracy in the recall and true–false tasks was positively associated with reading comprehension, and reaction time in the true–false task was negatively correlated with reading comprehension, while it was not significantly associated with reading comprehension in the recall task.

Previously, associations between reading comprehension, decoding, vocabulary, and working memory have been observed in child samples aged 8–12 years (Nouwens et al. 2017; Swart et al. 2017; Goff et al. 2005; Seigneuric et al. 2000) and in adolescent samples aged 12–15 years (Oslund et al. 2018). Additionally, these associations have been observed in adolescents in grades 6–12 (Arrington et al. 2014). The results of the current study, conducted with Russian-speaking adolescents aged 13–17 years, align with the results of previous investigations (Arrington et al. 2014; Oslund et al. 2018).

Our findings, such as the relationships between reading comprehension scores and decoding, vocabulary, and verbal working memory, are consistent with the SVR model (SVR; Gough and Tunmer 1986; Hoover and Gough 1990), which posits that reading comprehension is a multi-component process encompassing decoding, linguistic comprehension, and memory.

It is worth noting the specificity of the recall task and the true–false task. In the recall task, participants were required to remember whether a specific word was mentioned in the text they read; thus, this task aimed to assess word recognition. In the true–false task, participants made decisions about the semantic correctness of statements based on the text they had read, i.e., the level of understanding of the text they had read was determined.

The recall task. The better participants performed in the reading comprehension task, the more accurately they recognized the words in the recall task, supporting the contribution of working memory in the reading comprehension process (Daneman and Carpenter 1980; Just and Carpenter 1992; Swanson and Berninger 1995). Reading comprehension scores were not significantly correlated with the speed of recall, suggesting that the speed of recall may play a relatively minor role in reading comprehension.

The true–false task. The better participants performed in the reading comprehension task, the more accurately participants made decisions on the correctness of the statements in the true–false task. This suggests that both the true–false task and the reading comprehension task assess the same underlying construct: reading comprehension. Also, the better the participants performed in the reading comprehension task, the faster the participants made decisions on the correctness of the statements. This may indicate the contribution of reading speed to the reading comprehension process (e.g., Klauda and Guthrie 2008; Kuhn and Stahl 2003).

### 4.2. Eye Movement Data Results

By applying scanpath analysis to text reading data, we identified two distinct clusters of scanpaths. Within each cluster, the scanpaths had similar eye movement parameters, while among themselves, the clusters differed in these same parameters. The results indicate that cluster 1 is characterized by faster information processing with fewer rereadings of the text, while cluster 2 describes situations where participants needed more time to process the information. Therefore, we will henceforth refer to the two clusters of scanpaths as the “fast group” and “slow group.” Further analysis showed that the probability of using strategies from the fast group increased with increasing reading comprehension level and decreased for strategies from the slow group.

These findings are in line with previous research that also reported group separation among readers based on eye-movement patterns (Vorstius et al. 2014; Inhoff et al. 2016; Hyönä et al. 2002; Parshina et al. 2022a). For example, Inhoff et al. (2016) referred to readers with poorer comprehension levels as “slow readers” with fewer regressions. Hyönä et al. (2002) categorized readers into four clusters based on their eye movement characteristics where the group with high comprehension scores used fast forward saccades with minimal rereading, while the group with the worst comprehension and reading abilities exhibited a tendency towards slow, non-selective reading with frequent rereading of statements during the first read. Parshina et al. (2022a) observed that readers who tended to use the identified “fluent pattern” were characterized by direct reading from left to right, short fixation durations, a high probability of skipping words, and an absence of lengthy regressions and rereading of statements.

Our findings partially align with previous research, showing that reading comprehension proficiency can be characterized by specific scanpaths. The absence of significant differences in rereading time, number of regressions, and gaze duration per word characteristics during the first reading phase between fast and slow reading groups, in contrast to the presence of statistically significant differences in these parameters during the entirety of the text reading process, presents a particularly intriguing observation. The lack of group differences in parameters related to first-pass reading suggests that both groups engaged with the text with comparable levels of attention. However, significant differences emerged during the rereading stage. Considering that the accuracy of responses in the recall and true–false tasks is positively correlated with reading comprehension and that higher levels of comprehension are associated with an increased likelihood of using eye movement patterns indicative of fast reading, it can be concluded that fast readers are able to achieve sufficient comprehension and retain necessary details from the first reading, minimizing the need for subsequent rereading. Several studies describe regressive movements back in the text as a necessary part of improving reading comprehension (for example, Hyönä et al. 2002). Based on this, we can hypothesize that our results suggest that adolescents with less developed reading comprehension skills used regression to improve their understanding of the text.

An additional explanation for these findings may be related to the specific characteristics of the experimental task. The recall task, which followed every two paragraphs in the experimental procedure, required participants to remember well which words were used in the text previously. Such a task obviously influenced the participants’ potential use of a specific scanning reading pattern, prompting them to read words carefully upon first acquaintance with the text for both groups, as this could affect the successful completion of the recall task. From this perspective, a higher number of regressive movements back in the text for the slow group of readers may be associated not only with addressing comprehension issues but also with attempts to better memorize specific parts of the presented text. Previous research has demonstrated a strong association between reading and working memory (see review by Peng et al. 2018). However, the relationship between regressions and working memory is not straightforward. While Tanaka et al. (2014) proposed a dynamic interaction between internal working memory representations and regressions during reading, alternative explanations have emerged. Southwell et al. (2020), drawing upon the work of Meseguer et al. (2002), suggested that comprehension repair can occur covertly without observable changes in eye movements, potentially through reprocessing within working memory. Booth and Weger (2013) further posited that regressions may be driven by simple rereading rather than active memory, which focuses on specific text segments. They argued that under a high working memory load, readers might find it easier to reread the text to achieve task success, especially when the text is readily available, rather than relying on memory recall and processing after the first reading. Our findings suggest that active rereading constitutes a significant differentiating feature of the slow reading group. However, the specific factors contributing to this observed pattern remain unclear and require further investigation.

In previous research, it has been shown that readers with poorer reading skills exhibit smaller saccade amplitudes and higher skipping rates (Huestegge et al. 2009; Seassau and Bucci 2013; Sperlich et al. 2015). It has also been suggested that saccade amplitude may interact with a perceptual span, which refers to the amount of information extracted during a single fixation from the foveal and parafoveal zones. Importantly, the size of this effective reading field varies depending on the reading skills of readers of different ages (Marx et al. 2016; Veldre and Andrews 2014). In turn, changes in perceptual span during reading can influence saccade amplitude and vary depending on the level of reading proficiency (Huestegge et al. 2009; Veldre and Andrews 2014). However, as mentioned above, the specific nature of the task may have influenced the use of a strategy where fixation on each word could be necessary for readers to successfully complete the subsequent task. In such a case, the lack of differences between the groups of fast and slow readers in terms of saccade amplitude appears to be consistent with this observation.

Differences in oculomotor performance between groups are primarily related to the frequency of regression, with more regression occurring in the “slow” group. Differences are also observed in the speed of textual information processing, where the ‘fast’ group completed the task in a shorter period of time. The reason for the regressions can be attributed to the necessity of repeated reading to improve comprehension, which in turn increases the time taken to read and comprehend the text. However, the recall task may also influence the eye movement pattern while reading used by both groups. Thus, it can be concluded that the highlighted groups of readers exhibited different patterns of eye movements during the reading of the paragraphs, and these differences reflect the general level of reading comprehension. These findings partially align with data obtained previously in research conducted on different stimuli. However, testing hypotheses about eye movement patterns reflecting reading comprehension requires research aimed at studying natural reading under more ecological conditions.

## 5. Limitations and Future Directions

Although this study contributes to the field, it has a number of limitations. Thus, the main limitation of this study relates to the experimental design itself. As mentioned previously, the design of this study, specifically the use of recall following each two paragraphs of the text, might have influenced or modulated reading strategies. Participants may have adopted a specific reading pattern within the task, requiring detailed reading for successful completion.

Beyond reading itself, eye movement patterns are influenced by other factors that we did not control for. Reading goals, text genre, and structure can act as additional factors influencing reading strategies and, subsequently, comprehension. Previous studies have shown that eye movement activity and reading strategies adapt under the influence of certain task types (Kaakinen and Hyönä 2010; Strukelj and Niehorster 2018), but research in this area is limited, and the influence of tasks on comprehension and strategies is not fully understood. Future research in this area is needed to determine the role and contribution of all these factors in the process of understanding what is read and extracting information from text.

## Figures and Tables

**Table 1:** Means, standard deviations, minimal and maximal values, and Pearson’s correlations for the variables of interest.

Variable	Mean	SD	Min	Max	1	2	3	4	5	6	7
1. RC	29.79	10.59	8	46	1						
2. WS	55	9.60	26	74	0.591 **	1					
3. PS	20.96	6.99	6	34	0.717 ***	0.801 ***	1				
4. SYN	52.71	18.40	10	77	0.769 ***	0.725 ***	0.809 ***	1			
5. WD	40.29	2.64	32	42	0.579 **	0.546	0.380 *	0.686 ***	1		
6. PD	17.32	2.86	9	20	0.439 *	0.430 *	0.308	0.450 *	0.632 ***	1	
7. Age (years, month)	15.71	1.56	13.16	17.93	0.348	0.297	0.277	0.473 *	0.297	0.382 *	1

Note. RC = reading comprehension, WS = word span, PS = pseudoword span, SYN = synonyms, WD = word decoding, PD = pseudoword decoding. * *p* < .05, ** *p* < .01, *** *p* < .001.

**Table 2:** Descriptive statistics for the eye movement measures of two clusters of scanpath reading processes.

	Cluster 1 Mean (SD)	Cluster 2 Mean (SD)
Total reading time (word, ms)	387.23 (249.78)	515.05 (319.21)
Total time reading (paragraph, s)	33.01 (11.57)	51.51 (22.92)
Average fixation duration, (word, ms)	215.05 (78.81)	229.19 (86.52)
Average number of fixations (word)	1.74 (1.03)	2.16 (1.3)
Average saccade amplitude	2.8 (3.42)	2.26 (3.03)
Average number of regressions	0.19 (0.39)	0.2 (0.4)
Skip rate	0.5 (0.5)	0.5 (0.5)
Rereading time during first-pass (ms)	173.19 (668.47)	217.43 (760.64)
Average number of regressions during first-pass	0.11 (0.32)	0.12 (0.33)
Gaze duration during the first pass (word, ms)	293.93 (157.89)	345.66 (192.67)

Note: Skip rate = average number of words that were skipped during the reading process; SD = standard deviation.

**Table 3:** Linear mixed-effects model outputs for eye movement measures of the difference between scanpath clusters.

	Estimates	95% CI	t-Value	*p*-Value
1. Average duration of fixation (ms)				
Intercept	219.45	210.92, 227.99	50.39	.001
cluster 2	4.18	2.22, 6.15	4.17	.001
2. Total reading time per word (ms)				
Intercept	421.30	383.03, 459.63	21.56	.001
cluster 2	27.09	17.50, 36.67	5.54	.001
3. Average number of fixations per word				
Intercept	1.84	1.70, 1.97	26.94	.001
cluster 2	0.10	0.06, 0.14	4.79	.001
4. Average saccade amplitude				
Intercept	2.68	2.39, 2.97	18.00	.001
cluster 2	0.001	−0.08, 0.08	0.03	0.98
5. Total number of regressions				
Intercept	1.36	1.28, 1.43	12.95	.001
cluster 2	0.05	0.03, 0.07	2.74	.006
6. Total reading time for paragraph (s)				
Intercept	36.25	30.6, 41.91	12.62	.001
cluster 2	6.49	4.58, 8.4	6.68	.001
7. Average skip rate				
Intercept	0.51	0.43, 0.59	12.73	.001
cluster 2	−0.005	−0.02, 0.007	-0.81	0.421
8. Rereading time during first-pass reading (ms)				
Intercept	193.69	155.93, 231.46	10.05	.001
cluster 2	15.68	−16.91, 48.26	0.94	0.346
9. Number of regression during first-pass reading				
Intercept	0.12	0.10, 0.13	13.30	.001
cluster 2	0.007	−0.008, 0.02	0.91	0.361
10. Gaze duration per word during first-pass reading (ms)				
Intercept	337.44	289.98, 384.89	13.94	.001
cluster 2	7.60	−4.49, 19.69	1.23	0.22

Note: We built a separate model for each eye movement parameter. In this table, we have presented results only for the fixed factors of each model. Models numbered 1–7 reflect the variables of the entire reading period; models numbered 8–10 reflect the reading process during the first pass. Each model includes clusters as a fixed factor. A categorical fixed factor with a dummy code was used for each model. The intercept represented the result of cluster 1. Participant and trial intercepts were included as random factors in each model.

**Table 4:** Logistic mixed-effects model outputs for estimating the likelihood of using a specific cluster of the scanpath based on a reading comprehension score.

		Cluster 1			Cluster 2	
	Estimates	SE	*p*-Value	Estimates	SE	*p*-Value
Intercept	5.31	2.18	0.015	−5.31	2.28	0.02
RC	0.98	0.29	0.0001	−0.98	0.29	0.0001
Age	−0.27	0.14	0.055	0.27	0.15	0.07

Note. We built a separate model for each cluster. Both models include the mean-centered reading comprehension score and mean-centered age as the fixed factors. The intercept represents the mean-centered value of reading comprehension and age. Random intercepts of participants and trials were included as random factors.

## Data Availability

The data and analysis code for this study can be obtained by contacting the corresponding author.

## Supplementary Materials

The following supporting information can be downloaded at https://www.mdpi.com/article/10.3390/jintelligence12110112/s1: Figure S1: A scheme of the design of the reading skill assessment block. A fixed sequence was used, and all tasks were conducted one-on-one with an assessor; Table S1: The participants’ demographic characteristics and reading habits; Table S2: Language environment of the participants; Table S3: Logistic mixed-effects model outputs for response accuracy of the recall and true–false task depending on the reading comprehension score and the participant age; Table S4: Linear mixed-effects model outputs for the response time of the recall and true–false task depending on the reading comprehension (RC) score and the participant’s age.
