# Supplementary material for: Eye Movement Patterns in Russian-Speaking Adolescents with Differing Reading Comprehension Proficiency: Exploratory Scanpath Analysis
Source: J Intell. 2024 Nov 5;12(11):112. doi: 10.3390/jintelligence12110112 (PMC11595877; doi:10.3390/jintelligence12110112)
Supplement: Supplementary file 1 [file jintelligence-12-00112-s001.zip › jintelligence-3264030-supplementary.pdf]

Supplementary Materials

Table S1. The participants' demographic characteristics and reading habits.

**Variable**		*n*	%
Age			
	13 years	7	25
	14 years	10	35.71
	15 years	2	7.14
	16 years	4	14.29
Grade			
	6th	1	3.57
	7th	3	10.71
	8th	11	39.29
	9th	2	7.14
	10th	4	14.29
	11th	7	25
Gender			
	Male	22	78.57
	Female	6	21.43
Daily reading time (% of the typical day)			
	<10%	4	14.29
	from 10% to 20%	5	17.86
	from 20% to 50%	11	39.29
	>50%	8	28.57
Leading arm			
	Left	2	7.14
	Right	26	92.86

Table S2. Language environment of the participants.

Language Environment	*n*	%
Home		
Russian language	26	92.86
Other language	0	0
Russian and Other Language	2	7.14
School		
Russian language	22	78.57
Other language	0	0
Russian and Other Language	6	21.43
Free time		
Russian language	25	89.29
Other language	0	0
Russian and Other Language	3	10.71
Social interaction		
Russian language	27	96.43
Other language	0	0
Russian and Other Language	1	3.57
Experience of living in non-Russian speaking environment		
yes	5	17.86
no	23	82.14

Table S3. Logistic Mixed-Effects Model outputs for response accuracy of the recall and true-false task depending on the reading comprehension score and the participant age.

	estimates	*95% CI*	*z-value*	*p-value*
**Recall**				
Intercept	1.380	1.089, 1.683	9.578	.001
RC	0.260	0.094, 0.426	3.195	.001
Age	0.064	-0.105, 0.233	0.772	.440
**True-false**				
Intercept	1.691	1.266, 2.142	7.732	.001
RC	0.409	0.096, 0.732	2.633	.008
Age	0.170	-0.153, 0.489	1.080	.280

Note. Both models include the mean-centered reading comprehension (RC) score and mean-centered age as the fixed factors. The intercept represents the mean-centered value of reading comprehension and age. Random intercepts of participants and trials were included as random factors.

Table S4. Linear mixed-effect model outputs for the response time of the recall and true-false task depending on the reading comprehension (RC) score and the participant's age.

	estimates	*95% CI*	*t-value*	*p-value*
**Recall**				
Intercept	1908.08	489.424, 3327.407	2.728	.011
RC	-89.84	-232.065, 52.388	-1.282	.210
Age	-25.38	-118.364, 67.571	-0.554	0.584
**True-false**				
Intercept	2553.96	-2518.962, 7629.149	1.021	.316
RC	-587.22	-1096.164, -78.395	-2.342	.027
Age	112.55	-220.065, 445.049	0.687	.498

Note. Both models include the mean-centered reading comprehension (RC) score and mean-centered age as the fixed factors. The intercept represents the mean-centered value of reading comprehension and age. Random intercepts of participants and trials were included as random factors.

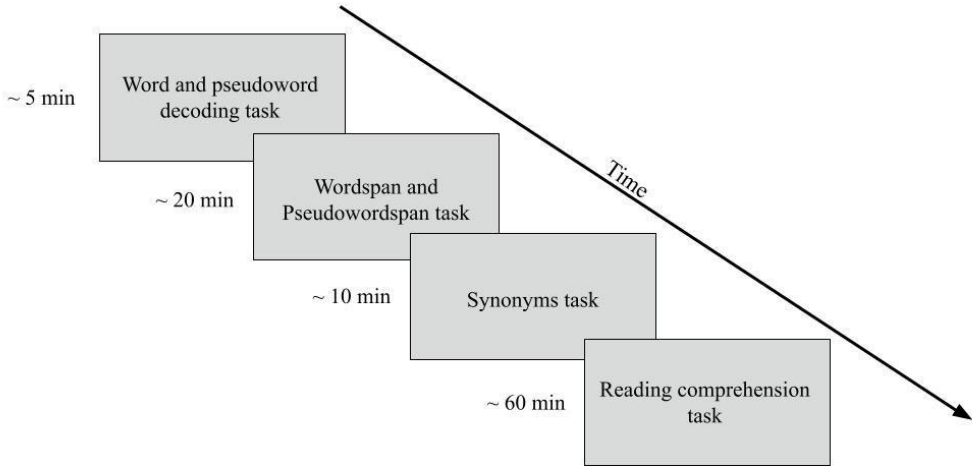

Figure S1. A scheme of the design of the reading skills assessment block. A fixed sequence was used, and all tasks were conducted one-on-one with an assessor.
